# State-of-the-Art Digital Imaging in Dentistry: Advanced Research of MRI, CT, CBCT, and Digital Intraoral Imaging

**DOI:** 10.1155/2018/9057120

**Published:** 2018-05-08

**Authors:** Eiichi Honda, Jerry L. Prince, Vania R. C. Fontanella

**Affiliations:** ^1^University of Tokushima Graduate School, Tokushima, Japan; ^2^Department of Electrical and Computer Engineering, The Johns Hopkins University, Baltimore, MD, USA; ^3^Dental School, Federal University of Rio Grande do Sul, Porto Alegre, RS, Brazil

State-of-the-art refers to the cutting-edge development in a particular field, bringing the newest evidence and representing the current and emerging trends and research priorities.

Diagnostic imaging in dentistry has seen enormous development in the last two decades. In spite of the compelling research interest in Cone Beam Computed Tomography (CBCT) [1], Magnetic Resonance Imaging (MRI), having no ionizing radiation exposure, has also shown significant potential to clarify relevant clinical questions [2]. Still, intraoral radiographs are the most common imaging method used in dental practice. The shift from analog to digital imaging was the earliest revolution in modern dentomaxillofacial radiology, bringing many advantages to the clinical work flow [3]. However, there is yet need for further development of the digital receptors.

For this special issue, we received many papers from all over the world, and the Guest Editors team selected five high-quality and peer-reviewed articles that addressed major aspects related to these modalities.

One of these studies reports a systematic review that investigates CBCT to visualize the upper airway before and after rapid maxillary expansion in growing patients. Authors pointed the need for a consistent protocol with regard to patient positioning and volume segmentation. CBCT was also proven to be accurate for bone measurements in close proximity to titanium implants in an animal model study.

Regarding MRI, another animal model study evaluated the optimal time interval of repeated intravenous injections of iodixanol in order to minimize acute kidney injury.

Moreover, as the quality of images is mandatory for dental and maxillofacial applications, one scatter reduction method on the head phantom, and the real patient is proposed to improve CBCT reconstructions. Finally, the performance of intraoral digital detectors is reported, comparing charge-coupled device and photostimulable phosphor plate systems.

*Eiichi Honda* *Jerry L. Prince* *Vania R. C. Fontanella*
